# Seed-Roasting Process Affects Oxidative Stability of Cold-Pressed Oils

**DOI:** 10.3390/antiox8080313

**Published:** 2019-08-16

**Authors:** Maria Barbara Różańska, Przemysław Łukasz Kowalczewski, Jolanta Tomaszewska-Gras, Krzysztof Dwiecki, Sylwia Mildner-Szkudlarz

**Affiliations:** 1 Institute of Food Technology of Plant Origin, Poznań University of Life Sciences, 60-624 Poznań, Poland; 2 Department of Food Safety and Quality Management, Poznań University of Life Sciences, 60-624 Poznań, Poland; 3 Department of Biochemistry and Food Analysis, Poznań University of Life Sciences, 60-623 Poznań, Poland

**Keywords:** antioxidant activity, berry by-product, berry seeds oil, DSC, oxidation stability

## Abstract

The oxidative stability of vegetable oils mainly depends on their fatty acid composition, their degree of unsaturation, and the presence of compounds with antioxidant activity. This paper reports on the effects of the process of roasting oil seeds, prior to pressing them, on the basic characteristics of the oils produced and their oxidative stability. The differential scanning calorimetry (DSC) technique was used to study the process of oxidation of the oil samples in an oxygen-flow cell. Chromatographic analysis revealed that roasting the seeds increased the levels of chlorophyll and β-carotene in all the cold-pressed oils. Similar results were observed for the oil’s antioxidant activity, measured by the scavenging 2,2-diphenyl-1-picrylhydrazyl (DPPH) radical method. Our results also indicated that roasting seeds prior to pressing them for oil had a positive effect on the oil’s stability, as determined by the DSC method. This manifested in both the extension of oxidation induction time and the final oxidation time.

## 1. Introduction

Fats and oils are important parts of the human diet, and are mainly (80%) derived from plant sources [1]. Oilseeds, such as oilseed rape or sunflower, are usually used to produce oil. However, raw materials, such as nigella and camelina, have become increasingly popular. The processing of berries (such as strawberries, raspberries, and blackcurrants) into juices and purees usually removes seeds as a byproduct [2]. Currently, the main method of waste seeds management is to use them in the production of animal feed preparations or compost production without pretreatment. The use of waste seeds as a raw material for oil pressing can provide a way to utilize the growing amount of waste, while also allowing the production of new oils with interesting nutritional values [3,4]. Cold-pressed oils have high levels of polyunsaturated fatty acids (PUFAs), but it should be noted that the oxidation of PUFAs is the main reaction that decreases oil quality. Depending on the origin and type of the oil, these fats are characterized by a range of oxidation stability, which can be expressed as the time necessary to produce and detect byproducts of oxidation. It is worth noting that this also depends on the storage conditions. This time is defined as the time of induction, beyond which a significant increase in the rate of lipid oxidation is observed [5]. Bioactive compounds present in oils, such as carotenoids, polyphenols, phytosterols, and tocochromanols, show a strong antioxidant effect that can protect against PUFA oxidation [6,7,8]. In addition, research results have shown the possibility of increasing the oxidative stability of oils by roasting the seeds prior to the pressing process [9,10]. Therefore, it was assumed that the roasting of seeds prior oil pressing might increase the oxidative stability of obtained oils, as measured by differential scanning calorimetry (DSC), and at the same time, antioxidant compounds will protect valuable nutritionally unsaturated fatty acids from deleterious changes.

There are many methods for measuring lipid oxidation, the most popular of which include the Rancimat, peroxide value, anisidine value, gas chromatography (GC), and nuclear magnetic resonance (NMR) methods [11]. Assessment of oxidative stability is increasingly carried out using the DSC test, which is a thermoanalytical technique [6,12,13]. Because of the measurement’s speed, as well as the fact that no toxic chemicals are required, DSC can also be successfully used to analyze the stability of oils in industry. The aim of this study was thus to use DSC to evaluate and compare the oxidative stability of different cold-pressed oils that had undergone seed roasting while chemically characterizing the oils and determining their antioxidant activity.

## 2. Materials and Methods 

### 2.1. Experimental Material and Oil Pressing

Nigella (SemCo, Śmiłów, Poland), winter and spring camelina (SemCo, Śmiłów, Poland), strawberry (GreenField, Warsaw, Poland), raspberry (GreenField, Warsaw, Poland), chokeberry (GreenField, Warsaw, Poland), blackcurrant (GreenField, Warsaw, Poland), and rapeseed (Swadzim Experimental Station, Swadzim, Poland) were roasted for 15 min at 140 °C, following a previously described methodology [14]. The oils were obtained by room-temperature pressing of the roasted (denoted “R”) and unroasted (“UR”) seeds using a Farmet Uno cold-pressing machine (Farmet, Česká Skalice, Czech Republic). After centrifugation for 15 min at 5000 rpm, the oils were stored under nitrogen in closed, fully filled bottles in the dark at 4 °C. The oils obtained from nigella, winter camelina, spring camelina, strawberry, raspberry, chokeberry, blackcurrant, and rapeseed are further denoted as BS, WC, SC, SB, RB, CB, BC, and R, respectively.

### 2.2. Chemical Characterization of Oils

Methyl esters of the fatty acids were characterized using the American Oil Chemists’ Society (AOCS) Ce 1h-05 method [15] on a Hewlett-Packard 5890 Gas Chromatograph equipped with a flame ionization detector and fitted with a Supelcowax-10 capillary column (30 m × 0.20 mm i.d. × 0.20 μm df, Supelco, Bellefonte, PA, USA). The fatty acids were identified by comparing their retention times with fatty acid methyl ester standards (Sigma-Aldrich, St. Louis, MO, USA) and the results were reported as weight percentages. 

Water content in oils was measured using the Karl Fischer method in accordance with ISO 8534 method [16].

Determination of chlorophyll content was performed following the AOCS Cc 13k-13 method [17], while the β-carotene content was determined spectrophotometrically using the calibration curve for β-carotene (Fluka, Buchs, Switzerland) (0.1–5 μg) dissolved in n-hexane, recording the absorbance at 440 nm [18]. 

The total tocochromanols content was determined using the method described by Siger et al. [19], injecting 200 mg of oil dissolved in high pressure liquid chromatography (HPLC)-grade n-hexane in a Waters Alliance HPLC System 600 (Waters, Milord, MA, USA) equipped with a LiChrosorb Si 60 column (250 × 4.6 mm; 5 μm, Merck, Darmstadt, Germany) and a fluorimetric detector (Waters 474). 

The calculated oxidizability (COX) of the oils was determined using the formula [20]:

$$COX\;Value=\;\frac{\left[\left(C18:1\;\left[\%\right]\right)+10.3\;\cdot \left(C18:2\;\left[\%\right]\right)+21.6\;\cdot \left(C18:3\;\left[\%\right]\right)\right]}{100}$$

### 2.3. Free Radical Scavenging Activity 

We measured the scavenging ability of the antioxidants in the oils in terms of the stable 2,2-Diphenyl-1-picrylhydrazyl (DPPH) radical [18]. One milliliter of seed oil was mixed with 1 mL of methanol in a PP tube (Sarsted AG & Co. KG, Nümbrecht, Germany) and shaken for 30 min, then centrifuged for 10 min at 700 g. The methanolic phase was collected and used for further tests. The antioxidant activity was tested using 20 μL of extract added to 3 mL of methanolic DPPH (0.04 mM). The mixture was allowed to stand for 60 min and absorption was measured at 517 nm against a reagent blank in a UV–vis spectrophotometer (Multiskan GO, Thermo Fisher Scientific, Vantaa, Finland). Results were expressed as μmol Trolox equivalent antioxidant capacity (TEAC) per 1 g of examined material.

### 2.4. Oxidative Stability of Pressed Oils Determined by DSC

A Perkin Elmer differential scanning calorimeter DSC 7 (Perkin Elmer, Norwalk, CT, USA), equipped with an Intracooler II and running under Pyris software, was used to examine the oxidative stability of the pressed oils. The equipment was calibrated using indium (m.p. 156.6 °C, ∆Hf = 28.45 J/g) and n-dodecane (m.p. −9.65 °C, ∆Hf = 216.73 J/g). Oil samples of 8–10 mg were weighed into open aluminum pans (Perkin Elmer, No. 02190041, Waltham, MA, USA), with an open empty aluminum pan serving as the reference. Prior to the oxidation analysis, purified nitrogen (99.99% purity) was passed at a flow rate of 20 mL/min. The determination of oxidation induction time (isothermal OIT) was carried out following the method described for oxidative stability measurements [21]. It was analyzed at least three times by isothermal heat flux at 140 °C under a constant oxygen flow of 20 mL/min. The oxidation induction time (t_OIT_) was determined by the tangent method as the intersection of the extrapolated baseline and the tangent (leading edge) to the recorded exotherm. Moreover, the oxidation final time (t_OFT_) was determined as the intersection of the tangent to the leading exotherm edge and the tangent to the increasing baseline after oxidation.

### 2.5. Statistical Analysis

All measurements were repeated three times, unless stated otherwise. For significant results, Tukey’s Significant Difference test was used. All tests were considered significant at *p* < 0.05. Statistical analysis was performed with Statistica 13 software (Dell Software Inc., Round Rock, TX, USA).

## 3. Results and Discussion

### 3.1. Fatty Acid Composition

Table 1 shows the average fatty acid composition of the examined oils. It can be seen that rapeseed oil contains predominantly oleic acid (C18:1 n−9). It was also characterized by the highest sum of monounsaturated fatty acids. However, from the point of view of oil stability and apart from the effect of native antioxidants and pro-oxidative substances, the autoxidation of oils is essentially determined by the type and quantity of their PUFA components [9]. The highest linoleic acid content (C18:2 n−6) was found in oil from chokeberry seeds (~74%). Other berry oils have a similar linoleic acid content of approximately 50%. In both rapeseed oil and winter and spring camelina seed oils, the linolenic acid content did not exceed 20%. It is worth noting that C18:2 n-6 fatty acid is 40 times more reactive than C18:1 n−9, and the oxidative capacity of each PUFA approximately doubles with each additional bis-allyl methylene group [22]. Equally important from the point of view of oxidative stability is alpha-linolenic acid (C18:3 n−3). Unlike other berry seed oils, the chokeberry seed oil contained only a negligible amount of this acid (0.6%)—10 times less than in the case of rapeseed oil, and almost 100 times less than in the other berry seed oils. Blackcurrant seed oil also contains gamma-linolenic acid (C18:3 n−6), which is not present in the other berry seed oils. It was also shown that roasting seeds before pressing them did not significantly affect the fatty acid composition, which is consistent with the research results on the roasting of seeds prior to oil extraction [9,19]. The oxidative stability of polyunsaturated fatty acids in oils due to roasting might be partly due to the formation of Maillard reaction products (MRPs), such as nitrogenous macromolecules–melanoidins, which have known antioxidant action and protect PUFA against oxidation. Therefore, it can be stated that MRPs were able to extend shelf-life of oil [23,24,25]. The calculated oxidizability (COX) value related to fatty acid composition was similar for the roasted and unroasted oil samples. Rapeseed UR and R oils had the lowest COX value of any of the examined oils, which can be explained by the high levels of monounsaturated fatty acids and of minor components that affect the stability of rapeseed oils. According to Xu et al. [26], the lack of significant differences between COX values indicates that the fatty acid composition is not a suitable parameter for evaluating an oil’s oxidative stability. Thus, they mentioned that the tocochromanol content might serve as a crucial indicator of the oxidative stability of cold-pressed oil.

### 3.2. β-carotene, Chlorophyll, and Tocochromanols Contents

The oxidative stability of oils depends on its levels of β-carotene, chlorophyll, and tocochromanols [27,28,29]. The levels of individual compounds in the obtained oils were thus analyzed. The results are presented in Table 2. During the refining phase of oil production, chlorophyll is almost completely removed due to the unattractive color it lends to the oil. It can also have pro-oxidative effects in the presence of light, so its level in edible oils should not exceed 50 mg/kg [30,31,32]. At the same time, Endo et al. [33] reported that chlorophyll and its degradation products (such as pheophytin) may have the ability to scavenge peroxyl and other free radicals, thus strongly inhibiting lipid oxidation during storage in soybean and rapeseed oils. Moreover, among all chlorophyll pigments, the majority of pheophytins are extracted into the oil during processing [34]. Three of the examined oils contained more chlorophyll than the permissible limit: BC/R (138.47 mg/kg), BC/UR (91.76 mg/kg), and SB/R (58.61 mg/kg). The lowest level of chlorophyll was recorded for BS/R and BS/UR (2.05 and 1.84 mg/kg, respectively). Carotenoid pigments, unlike chlorophyll, are desirable in oils due to their antioxidant properties and also contribute to the attractive yellow oil color [35,36]. The highest β-carotene level of over 100 mg/kg was seen in the oils from chokeberry seeds, blackcurrant seeds, and winter camelina, while the lowest levels were found in the oils from rapeseed and nigella. The higher levels of coloring pigments in the crude oils from roasted seeds may be explained by the fact that roasting increases the efficiency of extraction of chlorophyll and β-carotene, probably due to the rupturing of cell membranes [37]. It is worth noting that roasting of seeds prior oil pressing led to significant increase of water content in all analyzed samples. This could be explained by microstructural changes of plant seeds caused by high temperature during the roasting process [38]. On account of thermally induced denaturation of seed’s protein, the amount of water, which was added during conditioning process of seed to reach a final moisture of 9–10%, could not be bound inside of roasted seeds. Hence, it can be assumed that more amount of unbonded water might be present in cold-pressed oils from roasted seeds. Moreover, increased water content in analyzed oils did not affect the decrease in the oxidative stability of the oils. Tocochromanols are the most important natural antioxidants found in vegetable oils, though their antioxidant activity depends on their isomers and concentrations [39]. In addition, tocopherols in high concentrations may sometimes exhibit pro-oxidative activity in oils [40]. It was observed that roasting seeds significantly reduced the levels of tocochromanols in the resulting oils (Table 2). Siger et al. [19] also indicated that roasting rapeseeds prior to pressing results in a significant reduction in the level of each tocopherol homolog.

### 3.3. Antioxidant Activity

It is well-known that the main role of antioxidants in oils is to protect unsaturated fatty acids against autoxidation. There are many methods for assessing antioxidant properties, but the DPPH test is the simplest and most accurate method. For these reasons, it is commonly used to evaluate oils [41,42]. Table 2 shows the antioxidant activity results. It can be seen that roasting the seeds prior to cold-pressed significantly increased the antioxidant properties of the oils. Roasting the raspberry seeds thus caused the antioxidant capacity of the resulting oil to increase from 710 to 1068 μmol TE/g. An increase in phenolic extractability due to roasting might explain why higher levels of phenolic compounds are found in these oils. Moreover, our previously published results show that oil from roasted raspberry seeds was characterized by higher content ellagic acid than oil from unroasted seeds [14]. The total tocochromanol levels play a major role in the antioxidant activity of oils [43], but an important role is also played by β-carotene and phenolic compounds [44]. As shown in our earlier studies [14], berry seed oils have high levels of polyphenolic compounds, which may explain their high antioxidant capacity. Moreover, it is worth considering the potential interactions between different types of lipophilic and hydrophilic antioxidants, which lead to an increase or decrease in antioxidant activity due to the synergistic or antagonistic effects of antioxidants [45]. The increase in total antioxidant activity may also be due to new compounds that result from seed roasting, such as Maillard-type browning reaction products, which are responsible for the increased stability of the oils [46]. Maillard reaction products with potential antioxidant activity were also identified during the roasting of saffron seed [47] and cocoa beans [48].

### 3.4. Oxidation Induction Time Measured by DSC

Oxidation stability is an important factor that determines the potential use of oil in food production. Table 3 presents the results of the isothermal determination of oxidative stability of oils by DSC analysis. This method was used as an accelerated test of the oxidative resistance of oils by exposing samples to elevated temperatures in the presence of excess amounts of oxygen. In all the DSC analyses, a sharp decline was observed in the exothermic curve during the initiation of the oxidation process due to the increase in heat evolved during oxidation. Table 3 presents the results for the induction time (t_OIT_) of the oxidation of oils pressed from roasted and unroasted seeds. It can be observed that rapeseed oil from both roasted and unroasted seeds possessed the longest induction time, with the shortest being recorded for chokeberry (unroasted seeds). Generally, the longer the induction time, the higher the oxidative oil stability. The results also allow us to establish the effect of seed roasting in terms of delaying the oxidation process. It can be seen that roasting the seeds led to a significant extension of the oxidation stability period for the oils from chokeberry (229%), raspberry (42%), blackcurrant (35%), rape (25%), and nigella (5%) seeds. Such a fast increase in various oils’ oxidative stability as a result of roasting has also been described by Wijesundera et al. [9], Rękas et al. [49], Siger et al. [50], and Mazaheri et al. [51]. Roasting had no significant effect on the oxidation induction time of strawberry or camelina oils. 

Most researchers publishing in the subject of DSC oils oxidation consider only induction time parameter. However, significant differences were also observed in the part of the DSC curve after the induction time. Therefore, the next parameter was measured, i.e., oxidation final time (t_OFT_), also shown in Table 3. The roasting process significantly extended the oxidation process only in the case of raspberry, chokeberry, and rapeseed oils. According to Saldaña and Martínez-Monteagudo [52], it is a parameter that expresses the start of the termination process, as induction time (t_OIT_) determines the beginning of propagation. However, to find out whether roasting affects the duration of the oxidation process, the difference between t_OFT_ and t_OIT_ must be calculated, which is presented as the third parameter (Δt) in Table 3. Surprisingly, the reverse rule can be seen, i.e., for roasted seeds, the duration of the oxidation process was shorter than in the case of unroasted seeds. Only in the case of chokeberry seeds were the times for roasted and unroasted seeds similar. Summing up, it can be stated that roasting the seeds extends induction time and increases oxidative stability of oil. 

## 4. Conclusions

Our results clearly indicate that oils, especially those obtained from berry fruit seeds, are a good source of unsaturated fatty acids, β-carotene, and tocochromanols, which have a significant effect on antioxidant properties. Both pigments and unsaturated fatty acids affect oxidative stability. DSC analysis showed that roasting the seeds prior to pressing led to a significant increase in the oxidative stability of the oils. The information obtained in this study may contribute to the design of the cold-pressing process, helping to increase the stability of these novel oils and, as a consequence, allowing commercial use in various applications.

## Figures and Tables

**Table 1:** Unsaturated fatty acid composition of cold-pressed oils from unroasted and roasted seeds [% w/w].

Sample	C16:1	C18:1	C18:2	C18:3 n−3	C18:3 n−6	C18:4	C20:1	C20:2	C22:1	∑SFA	∑MUFA	∑PUFA	U/S	COX Value
SB/R	0.04 ± 0.01 ^e^	14.56 ± 0.10 ^d^	48.48 ± 0.07 ^d^	30.81 ± 0.05 ^d^	ND	ND	0.24 ± 0.01 ^e^	ND	ND	5.87 ± 0.12 ^d^	14.84 ± 0.17 ^f^	79.29 ± 0.20 ^b^	16.04 ± 0.22 ^c^	11.79 ± 0.07 ^b^
SB/UR	0.07 ± 0.02 ^d^	14.55 ± 0.09 ^d^	48.66 ± 0.10 ^d^	30.28 ± 0.08 ^d^	ND	ND	0.28 ± 0.02 ^e^	ND	ND	6.16 ± 0.14 ^cd^	14.90 ± 0.09 ^f^	78.94 ± 0.19 ^b^	15.23 ± 0.17 ^cd^	11.70 ± 0.11 ^b^
BC/R	0.06 ± 0.01 ^d^	12.78 ± 0.11 ^e^	48.40 ± 0.18 ^d^	12.14 ± 0.12 ^e^	16.80 ± 0.09 ^a^	2.60 ± 0.09^a^	0.36 ± 0.02 ^de^	ND	ND	6.86 ± 0.09 ^c^	13.20 ± 0.28 ^f^	79.94 ± 0.22 ^b^	13.58 ± 0.14 ^e^	11.36 ± 0.18 ^b^
BC/UR	0.06 ± 0.01 ^d^	13.43 ± 0.10^e^	48.15 ± 0.21 ^d^	11.93 ± 0.06 ^e^	16.19 ± 0.11 ^a^	2.45 ± 0.05^a^	0.78 ± 0.03 ^c^	ND	ND	7.01 ± 0.10 ^c^	14.27 ± 0.21 ^f^	78.72 ± 0.18 ^b^	13.26 ± 0.09 ^e^	11.17 ± 0.08 ^bc^
RB/R	0.09 ± 0.02 ^d^	11.33 ± 0.12 ^f^	52.61 ± 0.19 ^c^	33.01 ± 0.14 ^c^	ND	ND	0.17 ± 0.01 ^f^	ND	0.14 ± 0.01 ^c^	2.65 ± 0.04 ^e^	11.73 ± 0.07 ^h^	85.62 ± 0.17 ^a^	36.73 ± 0.25 ^a^	12.66 ± 0.11 ^a^
RB/UR	0.09 ± 0.03 ^d^	11.26 ± 0.07 ^f^	51.92 ± 0.20 ^c^	33.67 ± 0.21 ^c^	ND	ND	0.20 ± 0.02 ^ef^	ND	0.18 ± 0.02 ^c^	2.68 ± 0.06 ^e^	11.73 ± 0.06 ^h^	85.59 ± 0.11 ^a^	36.31 ± 0.16 ^a^	12.73 ± 0.06 ^a^
WC/R	0.08 ± 0.02 ^d^	12.70 ± 0.19 ^e^	17.74 ± 0.09 ^g^	40.16 ± 0.18 ^b^	ND	ND	13.73 ± 0.09 ^a^	2.19 ± 0.09^a^	3.49 ± 0.02 ^b^	9.91 ± 0.27 ^b^	30.00 ± 0.32 ^b^	60.09 ± 0.29 ^d^	9.09 ± 0.20 ^f^	10.63 ± 0.22 ^c^
WC/UR	0.06 ± 0.02 ^d^	12.18 ± 0.12 ^f^	17.27 ± 0.12 ^g^	42.39 ± 0.16 ^ab^	ND	ND	11.98 ± 0.11 ^ab^	2.01 ± 0.11^a^	4.71 ± 0.02 ^a^	9.40 ± 0.15 ^b^	28.93 ± 0.19 ^b^	61.67 ± 0.15 ^d^	9.64 ± 0.19 ^f^	11.06 ± 0.17 ^c^
SC/R	0.04 ± 0.01 ^e^	13.86 ± 0.21 ^e^	18.05 ± 0.07 ^f^	42.55 ± 0.11 ^ab^	1.55 ± 0.02 ^b^	ND	11.05 ± 0.09 ^b^	ND	3.45 ± 0.02 ^b^	9.45 ± 0.26 ^b^	28.40 ± 0.33 ^bc^	62.15 ± 0.22 ^d^	9.58 ± 0.23 ^f^	11.52 ± 0.19 ^b^
SC/UR	0.06 ± 0.01 ^d^	12.25 ± 0.10 ^ef^	18.19 ± 0.10 ^f^	43.80 ± 0.07 ^a^	1.76 ± 0.13 ^b^	ND	11.37 ± 0.09 ^b^	ND	3.69 ± 0.01 ^b^	8.88 ± 0.17 ^b^	27.37 ± 0.29 ^c^	63.75 ± 0.31 ^d^	10.26 ± 0.31 ^f^	11.84 ± 0.26 ^b^
CB/R	0.10 ± 0.01 ^cd^	19.29 ± 0.12 ^c^	73.36 ± 0.13 ^a^	0.57 ± 0.01 ^g^	ND	ND	0.15 ± 0.02 ^f^	ND	ND	6.53 ± 0.08 ^cd^	19.54 ± 0.31 ^e^	73.93 ± 0.27 ^c^	14.31 ± 0.22 ^de^	7.87 ± 0.04 ^d^
CB/UR	0.10 ± 0.03 ^c^	18.74 ± 0.15 ^c^	73.99 ± 0.22 ^a^	0.59 ± 0.01 ^g^	ND	ND	0.14 ± 0.01 ^f^	ND	ND	6.44 ± 0.11 ^cd^	18.98 ± 0.30 ^e^	74.58 ± 0.22 ^c^	14.53 ± 0.14 ^d^	7.93 ± 0.08 ^d^
BS/R	0.14 ± 0.03 ^c^	23.94 ± 0.09 ^b^	58.20 ± 0.09 ^b^	0.33 ± 0.01 ^g^	1.68 ± 0.09 ^b^	ND	0.16 ± 0.01 ^f^	ND	ND	15.55 ± 0.21 ^a^	24.24 ± 0.23 ^cd^	60.21 ± 0.18 ^d^	5.43 ± 0.06 ^g^	6.67 ± 0.09 ^e^
BS/UR	0.13 ± 0.04 ^c^	22.73 ± 0.10 ^b^	59.26 ± 0.11 ^b^	0.35 ± 0.02 ^g^	2.09 ± 0.03 ^b^	ND	0.18 ± 0.03 ^f^	ND	ND	15.26 ± 0.18 ^a^	23.04 ± 0.19 ^d^	61.70 ± 0.20 ^d^	5.55 ± 0.07 ^g^	6.86 ± 0.11 ^e^
R/R	0.30 ± 0.03 ^b^	70.01 ± 0.09 ^a^	19.65 ± 0.07 ^e^	6.68 ± 0.04 ^f^	ND	ND	0.43 ± 0.02 ^d^	ND	ND	2.93 ± 0.02 ^e^	70.74 ± 0.25 ^a^	26.33 ± 0.17 ^e^	33.13 ± 0.21 ^b^	4.17 ± 0.03 ^f^
R/UR	0.62 ± 0.03 ^a^	69.19 ± 0.11 ^a^	19.49 ± 0.08 ^e^	7.06 ± 0.09 ^f^	ND	ND	0.68 ± 0.03 ^c^	ND	ND	2.96 ± 0.02 ^e^	70.49 ± 0.22 ^a^	26.55 ± 0.18 ^e^	32.78 ± 0.26 ^b^	4.22 ± 0.05 ^f^

Mean values with different letters (^a–h^) in the columns are significantly different at α = 0.05. ∑SFA—sum of saturated fatty acids; ∑MUFA—sum of monounsaturated fatty acids; ∑PUFA—polyunsaturated fatty acids; U/S—unsaturated fatty acids to saturated fatty acids ratio; ND—not detected; SB/R, BC/R, RB/R, WC/R, SC/R, CB/R, BS/R, R/R—oils obtained from roasted seeds of strawberry, blackcurrant, raspberry, winter camelina, spring camelina, chokeberry, nigella, and rapeseed, respectively. SB/UR, BC/UR, RB/UR, WC/UR, SC/UR, CB/UR, BS/UR, R/UR—oils obtained from unroasted seeds of strawberry, blackcurrant, raspberry, winter camelina, spring camelina, chokeberry, nigella, and rapeseed, respectively.

**Table 2:** Water, β-carotene, chlorophyll, and tocochromanols contents, as well as antioxidant activity of oils.

Sample	Water Content [ppm]	β-carotene Content [mg/kg]	Chlorophyll Content [mg/kg]	Tocochromanols [mg/100g]	TEAC [μmol Trolox/g]
SB/R	811.75 ± 12.19 ^d^	75.46 ± 4.01 ^d^	58.61 ± 0.61 ^c^	43.83 ± 0.24 ^m^	293.2 ± 13.3 ^f^
SB/UR	693.95 ± 16.76 ^e^	46.37 ± 5.10 ^e^	35.51 ± 1.90 ^d^	47.62 ± 0.07 ^k^	215.8 ± 13.4 ^i^
BC/R	919.70 ± 15.84 ^c^	119.08 ± 2.65 ^b^	138.47 ± 6.10 ^a^	116.12 ± 0.19 ^d^	334.6 ± 8.8 ^e^
BC/UR	709.40 ± 14.95 ^e^	100.66 ± 2.11 ^c^	91.76 ± 3.05 ^b^	125.34 ± 0.15 ^c^	320.6 ± 59.0 ^e^
RB/R	707.67 ± 72.67 ^e^	97.45 ± 2.13 ^c^	13.66 ± 0.16 ^e^	172.91 ± 0.28 ^b^	1068.0 ± 14.4 ^a^
RB/UR	620.50 ± 66.24 ^f^	75.44 ± 2.47 ^d^	9.63 ± 0.67 ^ef^	235.37 ± 0.23 ^a^	710.3 ± 13.9 ^bc^
WC/R	617.33 ± 24.31 ^f^	102.36 ± 0.99 ^c^	13.12 ± 0.73 ^e^	61.02 ± 0.11 ^j^	299.2 ± 5.8 ^f^
WC/UR	597.46 ± 21.14 ^g^	94.53 ± 2.80 ^c^	10.88 ± 0.24 ^e^	62.52 ± 0.37 ^i^	234.4 ± 2.9 ^i^
SC/R	616.94 ± 27.33 ^f^	23.17 ± 0.40 ^f^	2.56 ± 0.08 ^fg^	63.85 ± 0.11 ^h^	241.3 ± 11.1 ^h^
SC/UR	603.83 ± 31.18 ^f^	22.96 ± 0.43 ^f^	2.03 ± 0.04 ^g^	68.13 ± 0.09 ^g^	198.7 ± 11.6 ^j^
CB/R	1114.10 ± 35.09 ^a^	147.58 ± 4.19 ^a^	7.69 ± 0.08 ^efg^	79.11 ± 0.58 ^f^	313.7 ± 2.9 ^e^
CB/UR	1020.50 ± 99.63 ^b^	114.42 ± 1.57 ^b^	7.38 ± 0.18 ^efg^	104.24 ± 0.04 ^e^	275.3 ± 11.7 ^g^
BS/R	401.31 ± 11.71 ^h^	10.24 ± 0.58 ^g^	2.05 ± 0.11 ^g^	17.61 ± 0.41 ^o^	737.4 ± 5.5 ^b^
BS/UR	371.12 ± 10.84 ^i^	9.72 ± 0.17 ^g^	1.84 ± 0.04 ^g^	25.42 ± 0.11 ^n^	670.7 ± 46.9 ^c^
R/R	714.15 ± 12.19 ^e^	16.10 ± 0.14 ^fg^	7.98 ± 0.05 ^efg^	45.74 ± 0.08 ^l^	432.6 ± 8.7 ^d^
R/UR	634.75 ± 37.97 ^f^	11.11 ± 0.16 ^g^	3.49 ± 0.08 ^fg^	46.84 ± 0.27 ^k^	251.9 ± 26.3 ^gh^

Mean values with different letters (^a–o^) in the columns are significantly different at α = 0.05 (*n* = 6). SB/R, BC/R, RB/R, WC/R, SC/R, CB/R, BS/R, R/R—oils obtained from roasted seeds of strawberry, blackcurrant, raspberry, winter camelina, spring camelina, chokeberry, nigella, and rapeseed, respectively. SB/UR, BC/UR, RB/UR, WC/UR, SC/UR, CB/UR, BS/UR, R/UR—oils obtained from unroasted seeds of strawberry, blackcurrant, raspberry, winter camelina, spring camelina, chokeberry, nigella, and rapeseed, respectively.

**Table 3:** Differential scanning calorimetry (DSC) oxidation induction time (t_OIT_), and oxidation final time (t_OFT_) of oils pressed from unroasted and roasted seeds.

Sample	t_OIT_ [min]	t_OFT_ [min]	Δt
SB/R	5.00 ± 0.12 ^a^	15.84 ± 0.15 ^b^	10.84 ^b^
SB/UR	5.12 ± 0.02 ^a^	18.12 ± 1.32 ^a^	13.00 ^a^
BC/R	17.76 ± 0.44 ^a^	24.07 ± 0.14 ^a^	6.31 ^b^
BC/UR	13.13 ± 0.08 ^b^	24.25 ± 0.21 ^a^	11.12 ^a^
RB/R	24.65 ± 0.12 ^a^	34.76 ± 1.28 ^a^	10.11 ^b^
RB/UR	17.40 ± 0.46 ^b^	30.87 ± 1.95 ^b^	13.47 ^a^
WC/R	9.98 ± 0.10 ^a^	14.96 ± 1.01 ^b^	4.98 ^b^
WC/UR	7.79 ± 0.43 ^a^	17.94 ± 0.39 ^a^	10.15 ^a^
SC/R	8.70 ± 0.75 ^a^	18.58 ± 0.63 ^b^	9.88 ^b^
SC/UR	8.96 ± 0.05 ^a^	22.74 ± 0.54 ^a^	13.78 ^a^
CB/R	10.88 ± 0.17 ^a^	26.59 ± 0.57 ^a^	15.71 ^a^
CB/UR	3.31 ± 0.49 ^b^	18.44 ± 0.04 ^b^	15.13 ^a^
BS/R	41.91 ± 0.49 ^a^	54.89 ± 0.83 ^a^	12.98 ^b^
BS/UR	39.89 ± 0.34 ^b^	54.05 ± 0.37 ^a^	14.16 ^a^
R/R	52.93 ± 1.74 ^a^	67.66 ± 0.35 ^a^	14.73 ^b^
R/UR	42.18 ± 2.33 ^b^	58.07 ± 3.42 ^b^	15.89 ^a^

Different letters (^a,b^) indicate significant differences between mean values for roasted and unroasted seed oils at α = 0.05. Δt = t_OFT_–t_OIT_ SB/R, BC/R, RB/R, WC/R, SC/R, CB/R, BS/R, R/R—oils obtained from roasted seeds of strawberry, blackcurrant, raspberry, winter camelina, spring camelina, chokeberry, nigella, and rapeseed, respectively. SB/UR, BC/UR, RB/UR, WC/UR, SC/UR, CB/UR, BS/UR, R/UR—oils obtained from unroasted seeds of strawberry, blackcurrant, raspberry, winter camelina, spring camelina, chokeberry, nigella, and rapeseed, respectively.
